# Nanopore Long‐Read Metagenomics Reveals Pollution‐Driven Antibiotic Resistance and Xenobiotic Degradation in Urban Beach Microbiomes

**DOI:** 10.1111/1758-2229.70396

**Published:** 2026-08-10

**Authors:** Angelo Felipe Barbosa de Oliveira, Bruno Santana Carneiro, Joelma Barbosa de Carvalho, Adan Rodrigues de Oliveira, Artur Luiz da Costa da Silva, Adonney Allan de Oliveira Veras, Rafael Azevedo Baraúna, Diego Assis das Graças

**Affiliations:** ^1^ Laboratório de Engenharia Biológica, Instituto de Ciências Biológicas, Universidade Federal do Pará Belém Pará Brazil; ^2^ Instituto Evandro Chagas Ananindeua Pará Brazil

## Abstract

Coastal ecosystems are vital for biodiversity but are increasingly threatened by urbanisation and pollution, which significantly alter local microbial communities. This study assessed bacterial diversity and functional profiles in urban and island beaches in Belém, Brazil. Urban beaches showed significantly higher microbial diversity and evenness, alongside functional plasticity due to pollutant input, while island beaches hosted more specialised and stable communities. Taxonomic analysis revealed the significant enrichment of opportunistic genera such as *Comamonas*, *Clostridium* and *Paenibacillus* in urban areas, and the massive dominance of *Prochlorococcus* and Candidatus *Pelagibacter* in island sites. Furthermore, shotgun metagenomics identified a robust genomic potential for xenobiotic degradation and antibiotic resistance in urban microbiomes, whereas island microbiomes were significantly enriched in genes for energy production and biosynthesis. These results underscore the ecological divergence between anthropogenically impacted and natural coastal environments, highlighting the importance of microbiome monitoring for sustainable coastal management.

## Graphical abstract

Using nanopore metagenomics, this study reveals that anthropogenic pollution drives ecological and functional shifts in coastal microbiomes. Pristine island environments act as stable, *Prochlorococcus*‐dominated baselines, whereas urban beaches function as hotspots for opportunistic taxa, harbouring a robust genomic potential for xenobiotic degradation and antibiotic resistance.

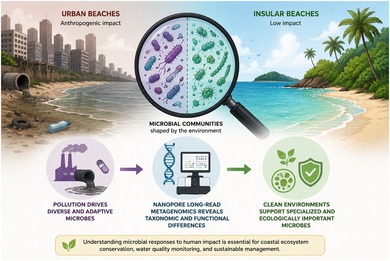

## 1 Introduction

Coastal ecosystems are dynamic environments where microbial communities are essential for ecological functions such as nutrient cycling, organic matter decomposition and biogeochemical stability (Zhi et al. 2023). Bacterial diversity drives these processes, influencing ecosystem resilience to natural and anthropogenic pressures. Urbanisation, pollution and climate change threaten microbial communities, emphasising the need for studies on how environmental factors affect microbial structure and function (Sharpe et al. 2023).

Urbanisation introduces pollutants like organic waste, heavy metals and emerging contaminants, which alter microbial diversity and community composition, often favouring stress‐resistant, opportunistic taxa. In contrast, insular environments, with less anthropogenic impact, provide baselines for understanding natural microbial organisation and function. Comparing microbial communities in these environments offers insights into microbial adaptation, resilience and ecological succession (Zhao et al. 2022; Miglani et al. 2022).

Alpha diversity metrics reflect the structure and complexity of microbial communities (Finn 2024). While overall species richness can remain stable across different coastal zones, urban beaches generally show significantly higher microbial diversity and evenness, driven by various microbial sources and environmental heterogeneity. Island beaches, however, harbour more specialised and stable communities, shaped by natural environmental gradients.

Beta diversity analyses, using techniques like principal coordinates analysis (PCoA) and redundancy analysis (RDA), highlight community composition differences between sites. These analyses reveal how environmental factors, such as pH, turbidity, dissolved oxygen and temperature, structure microbial assemblages, indicating niche differentiation between urban and island beaches (Liu et al. 2020).

Functional profiling reveals distinct metabolic strategies shaped by anthropogenic influences. Urban microbiomes typically show a higher genomic potential for xenobiotic degradation and antibiotic resistance, alongside specific metabolic categories (COGs) linked to rapid cellular turnover and adaptation (Cantalapiedra et al. 2021). Conversely, island microbiomes prioritise primary metabolic pathways for energy production, biosynthesis and nutrient cycling, reflecting a more stable ecological strategy.

The detection of pathogenic and opportunistic taxa, particularly in urban environments, raises concerns about coastal water safety and the spread of antimicrobial resistance genes. In contrast, island beaches host more phototrophic and nutrient‐cycling microorganisms, emphasising their ecological value in preserving natural microbial diversity (Liu et al. 2024).

This study aims to characterise bacterial communities in urban and insular beaches by integrating taxonomic, functional and environmental data. We seek to understand the impact of urbanisation on microbial community structure, identify environmental health bioindicators and explore the biotechnological potential of microbial consortia in contrasting coastal settings. This research contributes to environmental monitoring, conservation and sustainable use of microbial resources under increasing anthropogenic pressures (Alanzi 2024).

## 2 Materials and Methods

### 2.1 Study Sites and Sample Collection

**FIGURE 1:**
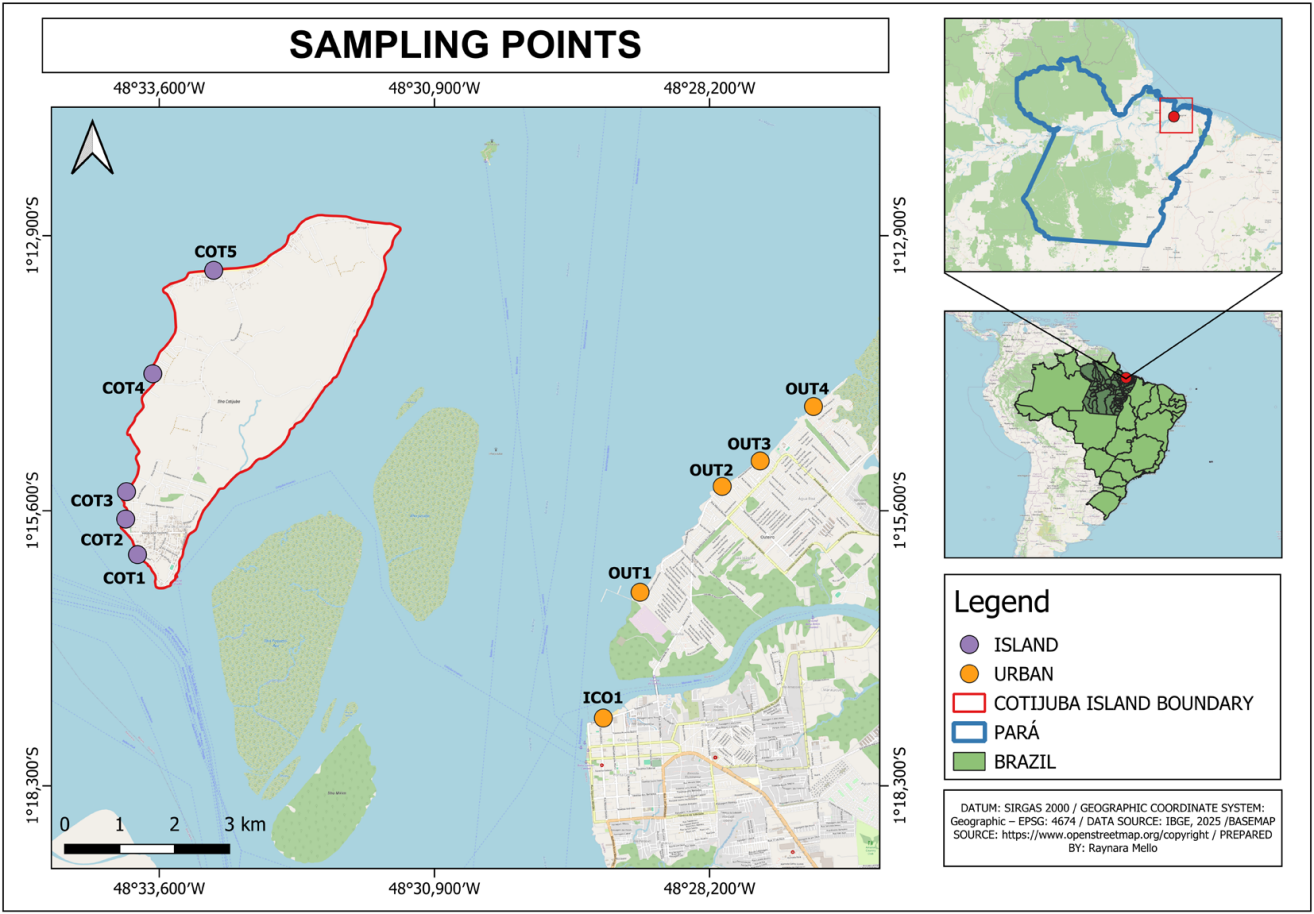
Map of the study sites and sampling locations.

Samples were collected in July 2024, with 10 beaches visited to obtain environmental surface water samples (Figure 1). The samples were collected using Whirl‐Pak Standard Sterilised Bags, with one sample collected per beach (*n* = 10), each containing 1 L. The urban districts were used for sample nomenclature (ICO for Icoaraci district, OUT for Outeiro district and COT for Cotijuba district). However, for analysis purposes, the samples were grouped into two categories: the urban group (Icoaraci and Outeiro districts) and the island group (Cotijuba district). At each location, 2 L of water was collected adjacently for physicochemical parameter analysis. Additional metadata, including the exact locations of the sampling points, as well as geographic, regional and climatic characteristics, were recorded.

### 2.2 Samples Processing

The samples were stored in temperature‐controlled containers (~4°C–6°C) during transport to the Biological Engineering Laboratory (ENGBIO, PCT Guamá) for genetic material extraction and biomolecular research, and to the Evandro Chagas Research Institute, environment section (SEAMB/IEC) for physicochemical analysis on the same day. The samples were processed upon arrival at the laboratory using the cold centrifugation method to precipitate material, followed by extraction of the precipitated material. Centrifugation was performed at 5000*g* for 5 min at 4°C. Although this centrifugation approach can potentially bias the recovered microbial fraction by leaving smaller or less dense cells in the supernatant, we experimentally evaluated this limitation by filtering the supernatant through a 0.22 μm membrane and performing DNA extraction with the same kit. No DNA was detected in this filtered supernatant fraction via Qubit quantification or agarose gel electrophoresis, indicating that the 5000*g* centrifugation effectively pelleted the relevant microbial biomass without significant loss. The supernatant was discarded, and the pellets were collected in sterile 15 mL Falcon tubes for subsequent DNA extraction. The SPINeasy DNA Kit for Microbiome (MP Biomedicals) was used for all samples following the manufacturer's instructions. The extracted DNA was stored at −20°C until 16S rDNA amplification by PCR and shotgun sequencing.

For each extracted DNA sample, the concentration of double‐stranded DNA (dsDNA) was determined using a Qubit fluorometer (Invitrogen, Waltham, MA, United States) with the Qubit dsDNA High Sensitivity Assay Kit and purity was measured with NanoDrop lite equipment (Invitrogen, Thermo Scientific, MA, United States). Amplification was performed with 5 ng of DNA for each sample, using amplicon PCR to target the full 16S rRNA gene with the forward primer 16S‐27F (5′‐AGA GTT TGA TCM TGG CTC AG‐3′) and the reverse primer 16S‐1492R (5′‐CGG TTA CCT TGT TAC GAC TT‐3′). Qubit dsDNA concentrations were measured again after the amplicon PCR.

Physicochemical analyses were conducted to assess water quality based on standard environmental monitoring practices. The variables pH, dissolved oxygen (DO), electrical conductivity, salinity, temperature and total dissolved solids (TDS) were measured in situ during sampling using a portable multiparameter probe (HI9829, HANNA, USA), previously calibrated according to the manufacturer's specifications.

Alkalinity was determined by volumetric titration, following standard titrimetric procedures. Turbidity was measured via spectrophotometry using a DR3900 spectrophotometer (HACH, USA). The concentrations of chloride (Cl^−^), nitrite (N‐NO_2_^−^), nitrate (N‐NO_3_^−^), sulphate (SO_4_^2−^), sodium (Na^+^), potassium (K^+^), calcium (Ca^2+^) and magnesium (Mg^2+^) were quantified by ion chromatography using a dual‐channel ICS3000DUAL system (Thermo Scientific/DIONEX, USA), operated according to the manufacturer's protocol for environmental water analysis.

### 2.3 Amplicon and Shotgun DNA Sequencing and Processing

For 16S sequencing, library preparation was conducted using the Ligation Sequencing Kit Amplicon—Native Barcoding Kit 96 V14 (SQD‐114.96) and Flowcell FLO‐PRO114M (Oxford Nanopore Technologies) using 200 ng of each amplicon, and the final quantity applied was 200 fmol. Raw sequence reads in FASTQ format were processed for quality control using FastQC (De Coster et al. 2018) prior to any assembly or alignment steps. Reads were filtered using Filtlong, removing those shorter than 1000 bp. Filtering by average quality was not necessary, as the native software of the sequencing platform (PromethION 2 Solo) already filters samples with a quality score above 10. Taxonomic classification was performed using the SILVA SSU 16S rRNA database 138.1 (Sayers et al. 2020). The taxonomic table, abundance table and phylogenetic tree were generated using Kraken2, Bracken, MAFFT and FastTree (Katoh and Standley 2013; Wood et al. 2019; Lu et al. 2017; Price et al. 2010).

These outputs, along with the metadata table, were imported into RStudio (R version 4.5.0) for downstream analysis using the phyloseq (version 3.2.1) and vegan (version 2.6‐10) packages (McMurdie and Holmes 2013; Oksanen 2024). Reads associated with unobserved taxa were excluded to refine the overall microbiome dataset. Taxonomic filtering was performed following the methods described by Callahan et al. (2016). To ensure the reproducibility of all analyses, a global random seed (123) was applied to all stochastic processes. Data quality and sampling sufficiency were verified using Good's Coverage with a minimum threshold of 0.95. Rarefaction curves were generated using a step size of 100 reads to evaluate species accumulation patterns. To account for differences in sequencing effort, the taxonomic abundance table was first normalised through rarefaction to the minimum library size.

Alpha diversity metrics (Observed OTUs, Chao1, ACE, Shannon, Simpson and Pielou's Evenness) were estimated using phyloseq and vegan. To assess differences in microbial community composition between urban and island beaches, beta diversity was evaluated using PCoA based on the Bray–Curtis dissimilarity matrix, chosen to account for both presence and relative abundance. Statistical significance of the observed clustering was determined through permutational multivariate analysis of variance (PERMANOVA) using the adonis2 function (999 permutations). For visualisation, 95% confidence intervals were represented by data ellipses.

To ensure the reproducibility of all analyses, a global random seed (123) was applied to all stochastic processes. Data quality and sampling sufficiency were verified using Good's Coverage with a minimum threshold of 0.95. Rarefaction curves were generated using a step size of 100 reads to evaluate species accumulation patterns. To account for differences in sequencing effort, the taxonomic abundance table was first normalised through rarefaction to the minimum library size.

For shotgun sequencing, the library preparation was conducted using the Ligation Sequencing gDNA—Native Barcoding Kit 96 V14 and Flowcell FLO‐PRO114M (SQK‐NBD114.96) (Oxford Nanopore Technologies) using 400 ng of each sample. Quality assessment of raw sequence reads was performed using FastQC (De Coster et al. 2018) prior to any assembly steps. Filtering by average quality was not necessary, as the native software of the sequencing platform (PromethION 2 Solo) already filters samples with a quality score above 10, and reads < 500 bp were filtered using Filtlong. Contig assembly was performed using the Flye v2.9.5 tool with the meta parameter, which is recommended for metagenomic data (Kolmogorov et al. 2020), and with Megahit v1.2.9 (Li et al. 2015). Subsequently, coverage files were generated using Minimap2 v2.28‐r1209 (Li 2018) and Samtools v1.16.1 (Danecek et al. 2021), by aligning the FASTQ sequences against the FASTA assemblies.

With the processed files, gene prediction was carried out using Prodigal v2.6.3 with the meta parameter (adjusted for metagenomes), followed by annotation using eggNOG‐mapper (Cantalapiedra et al. 2021; Huerta‐Cepas et al. 2018; Buchfink et al. 2021; Steinegger and Söding 2017), aiming to compare the abundance of functional categories between samples. For graphical visualisation of these differences, R packages such as ggplot2 were employed, along with functional enrichment analysis using GOseq and ClusterProfiler in the R environment to identify enriched functional categories in one sample compared to another.

### 2.4 Statistical Analysis

Data pre‐processing, including the filtering and normalisation of read counts, was performed prior to downstream evaluations to ensure accurate comparative analyses. Data are presented as relative abundances, proportions, or log2 fold changes, depending on the specific analysis. The total sample size for all statistical comparisons was *n* = 10 (comprising 5 urban and 5 island samples). Alpha diversity differences between the two environments were evaluated using the Kruskal–Wallis test (significance defined as *p* < 0.05). Beta diversity was assessed through PCoA based on Bray–Curtis dissimilarities, with statistical significance determined by PERMANOVA (*p* = 0.0090). A RDA was conducted to identify physicochemical drivers, evaluated via a Monte Carlo permutation test (*p* = 0.0060). Differential abundance was assessed using DESeq2 (*p* < 0.05). All statistical analyses and data visualisations were performed using the R software environment.

## 3 Results

### 3.1 Physicochemical Parameters in the Sampling Location

The median values of various physicochemical parameters measured at two distinct localities: Urban and Island. The table also includes the standard deviation (Std +/−) for each parameter, providing an overview of the variability within the respective samples (Table 1).

**TABLE 1:** Median of physicochemical parameters per locality.

Parameter	Urban	Std +/−	Island	Std +/−
Temperature (°C)	33.4	2.1	37.0	0
pH	6.8	0.15	7.5	0.1
Sample temperature	30	1	26.4	2.7
Conductivity (μS/cm)	416.2	36.3	1516.4	152.3
Dissolved total solids (mg/L)	208.2	17.8	738.2	35.4
Dissolved oxygen (mg/L)	5.8	0.1	4.0	0.1
Salinity (psu/ppt)	0.2	0.01	0.74	0.02
Turbidity (NTU)	41.1	6.6	66.7	4.5
Chloride (mg/L)	237.3	237.2	458.0	28.1
Nitrate (mg/L)	0.41	0.4	1.7	0.3
Nitrite (mg/L)	0.0	0	0.0	0
Sulphate (mg/L)	33.2	4.3	46.0	21
Sodium (mg/L)	201.0	60	371.9	57
Potassium (mg/L)	889.5	195	646.9	386
Magnesium (mg/L)	21.0	2.9	19.5	7.5
Calcium (mg/L)	10.0	1.1	11.2	1.9
Alkalinity (mg/L)	23.0	7.3	28.8	3.2

### 3.2 Data Output and Processing

Nanopore 16S rRNA sequencing generated high‐length reads, achieving an average read length of approximately 1580 bp and an average quality score of Q15. Following quality control, filtering and taxonomic classification, the overall microbiome dataset comprised a total of 8875 detected taxa across the 10 analysed samples. The detailed sequencing yield for both the 16S amplicon and shotgun metagenomic approaches across all samples is summarised in Table 2.

**TABLE 2:** Sample identification and sequencing yield.

Sample ID	Beach type	Amplicon reads	Shotgun reads	Shotgun yield per GB
ICO1	Urban	92.035	1.7M	2.4
OUT1	Urban	155.883	2.2M	3.1
OUT2	Urban	91.269	1.5M	2.1
OUT3	Urban	115.524	1.3M	2.1
OUT4	Urban	135.968	1.5M	2.0
COT1	Island	142.034	147K	0.293
COT2	Island	156.063	262K	0.643
COT3	Island	140.102	184K	0.471
COT4	Island	173.952	122K	0.293
COT5	Island	125.383	137K	0.281

### 3.3 Bacterial Diversity and Community Structure

#### 3.3.1 Alpha Diversity

Alpha diversity was evaluated to compare the microbial community structure between the island and urban environments (Figure 4). The analysis revealed no statistically significant differences in species richness between the two groups, as indicated by the metrics for Observed Richness (*p* = 0.7540), Chao1 (*p* = 0.6015) and ACE (*p* = 0.3472). In contrast, there were statistically significant differences in both species diversity and community evenness. Specifically, the urban environments exhibited significantly higher diversity and evenness compared to the island environments, which was consistently supported by the Shannon Index (*p* = 0.0090), Simpson Index (1 − *D*) (*p* = 0.0090) and Pielou's Evenness (*J*) (*p* = 0.0090).

#### 3.3.2 Beta Diversity

Beta diversity was evaluated using PCoA based on Bray–Curtis dissimilarity to assess differences in the microbial community composition between island and urban environments (Figure 5). The PCoA plot demonstrated a clear spatial separation and distinct clustering of samples according to their environment type along the primary axis. The first two principal coordinates accounted for a substantial majority of the data's variation, with PCoA 1 and PCoA 2 explaining 76.1% and 10% of the total variance, respectively. This visual separation was supported by statistical testing, as PERMANOVA confirmed a significant difference in the overall microbial community composition between the island and urban groups (*p* = 0.0090).

#### 3.3.3 Redundancy Analysis

RDA was performed to evaluate the influence of environmental drivers on the microbial community structure (Figure 6). The RDA model was statistically significant (*p* = 0.0060) and accounted for a substantial portion of the variation (adjusted *R* ^2^ = 0.604). The ordination plot revealed that island samples were positively correlated with salinity, turbidity and pH along the primary axes. Conversely, the urban samples clustered together on the opposite side of the axis and were primarily driven by factors such as dissolved oxygen and temperature.

#### 3.3.4 Overall Microbiome

**FIGURE 2:**
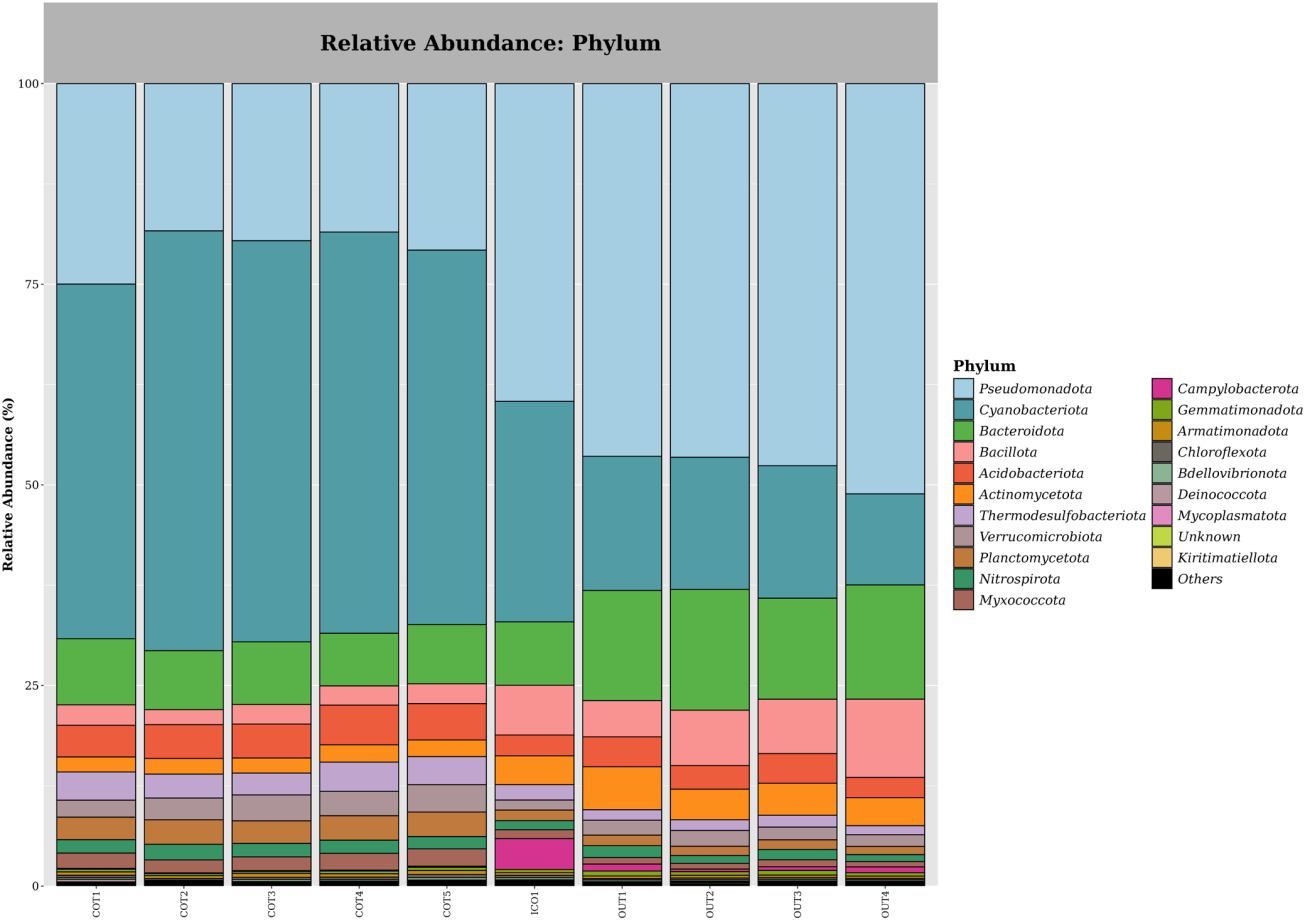
Relative abundance of bacterial communities at the phylum level across island and urban beaches. Sample size: *N* = 10 (5 urban, 5 island).

At the phylum level (Figure 2), the communities were primarily composed of Pseudomonadota, Cyanobacteriota and Bacteroidota. A distinct shift in community structure was observed between the two environments: Cyanobacteriota was highly prevalent in the island samples (mean relative abundance ~48.0%), whereas its relative abundance visibly decreased in the urban samples (~16.8%). Concurrently, the urban environments displayed an expanded proportion of Pseudomonadota (~46.6% vs. ~21.0% in the island), Bacteroidota (~13.2% vs. ~7.4%) and Bacillota (~7.2% vs. ~2.4%) compared to the island.

**FIGURE 3:**
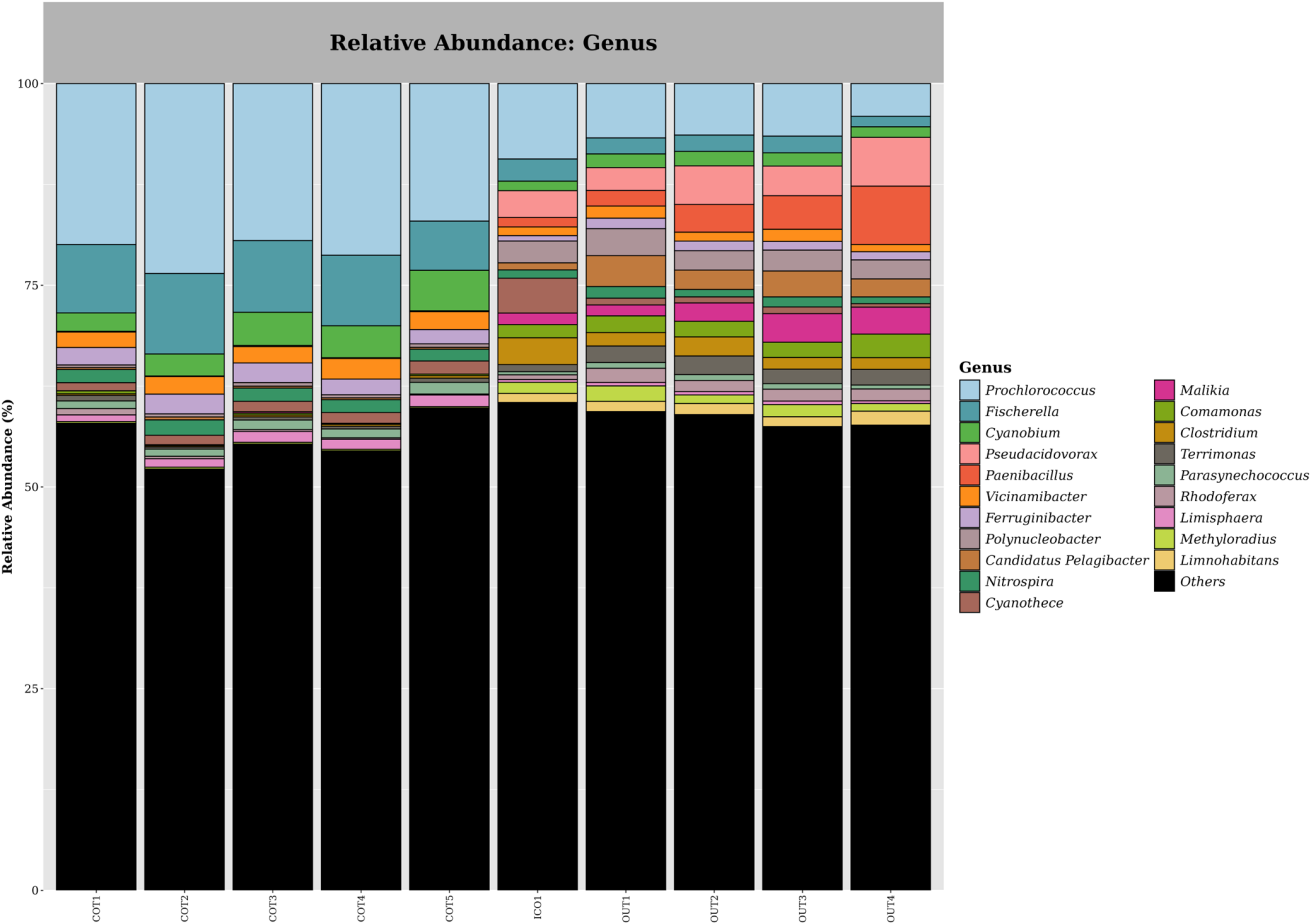
Relative abundance of bacterial communities at the genus level across island and urban beaches. Data are presented as relative sequence abundances (%). Sample size: *N* = 10 (5 urban, 5 island).

At the genus level (Figure 3), a substantial fraction of the sequences belonged to a diverse group of lower‐abundance taxa categorised as ‘Others’ across all samples (~55%–60%). Among the most highly abundant classified genera, *Prochlorococcus* was predominantly enriched in the island environment (~20%–30%), with its relative abundance markedly reduced in the urban samples (~5%–10%). In contrast, the urban samples exhibited a visible increase in the relative abundance of other genera, such as *Pseudacidovorax* (~5%–8% vs. < 1% in the island) and *Paenibacillus* (~2%–5% vs. < 1%).

**FIGURE 4:**
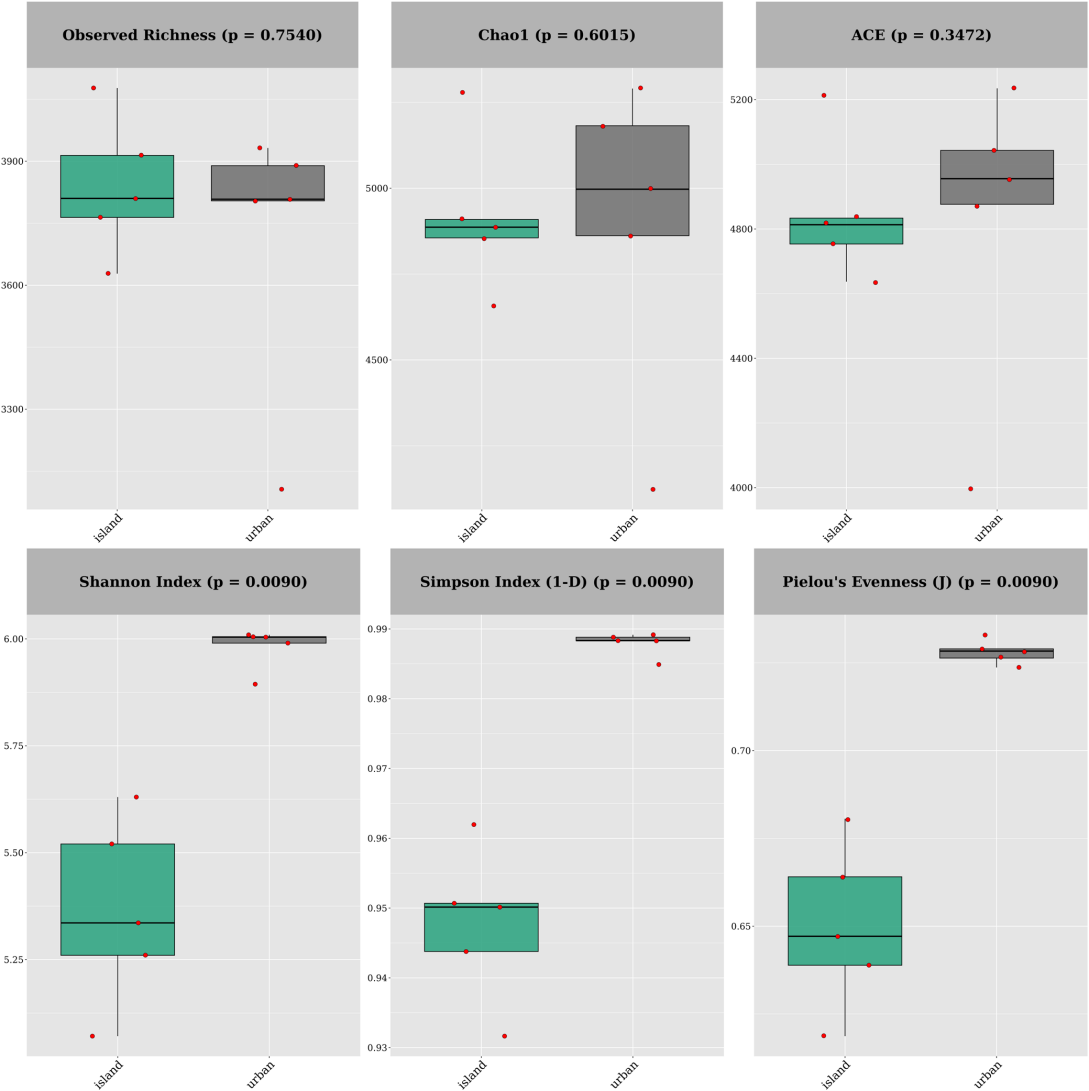
Alpha diversity metrics comparing microbial communities from island and urban environments. Statistical significance was assessed using the Kruskal–Wallis test. Sample size: *N* = 10 (5 urban, 5 island).

**FIGURE 5:**
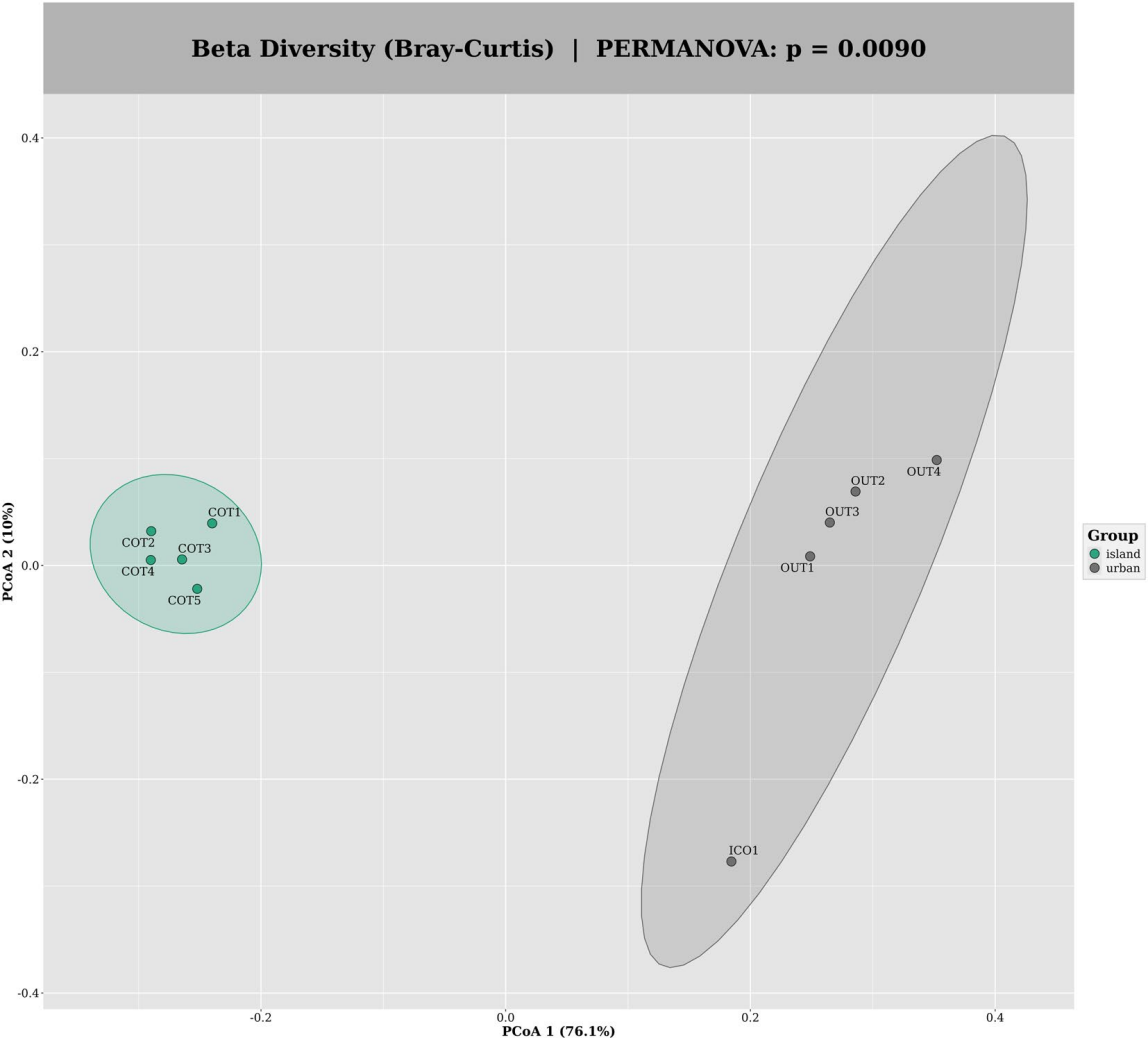
Principal coordinates analysis (PCoA) based on Bray–Curtis dissimilarity matrix. The statistical significance of the clustering between urban and island groups was determined by PERMANOVA (*p* = 0.0090). Sample size: *N* = 10.

**FIGURE 6:**
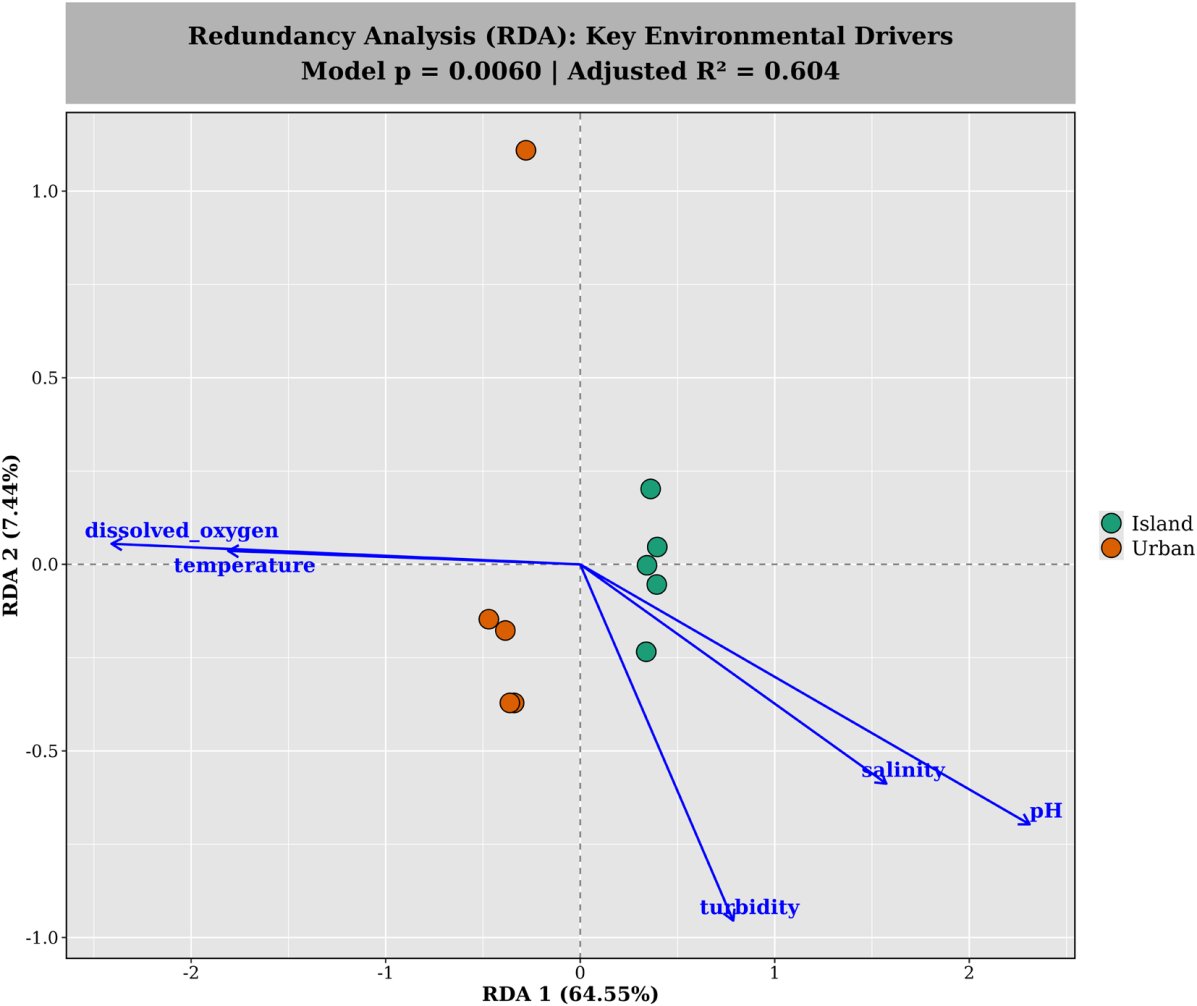
Redundancy analysis (RDA) illustrating the influence of key physicochemical parameters on microbial community structure. Model significance was assessed via Monte Carlo permutation test (*p* = 0.0060). Sample size: *N* = 10.

### 3.4 Differential Abundance

**FIGURE 7:**
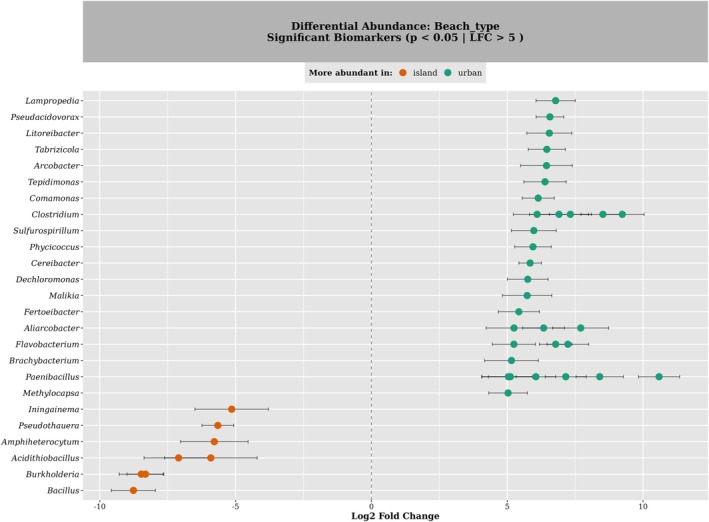
Differential abundance analysis of specific microbial biomarkers at the genus level. Significant taxa were identified using the DESeq2 package (adjusted *p* < 0.05). Sample size: *N* = 10.

To identify specific microbial biomarkers driving the differences between the environments and provide a concise overview of differential abundance, we performed a DESeq2 analysis focusing on highly significant taxa (*p* < 0.05 and absolute Log2 Fold Change > 5) (Figure 7). The analysis revealed that a substantially greater number of genera were significantly enriched in the urban environment compared to the island environment. Specifically, urban samples were characterised by strong positive fold changes for genera such as *Paenibacillus*, *Clostridium* and *Flavobacterium*. Conversely, the island environment exhibited significant enrichment in a smaller, distinct subset of taxa, most notably *Bacillus*, *Burkholderia* and *Acidithiobacillus*.

### 3.5 Functional Profiling and Metabolic Potential

**FIGURE 8:**
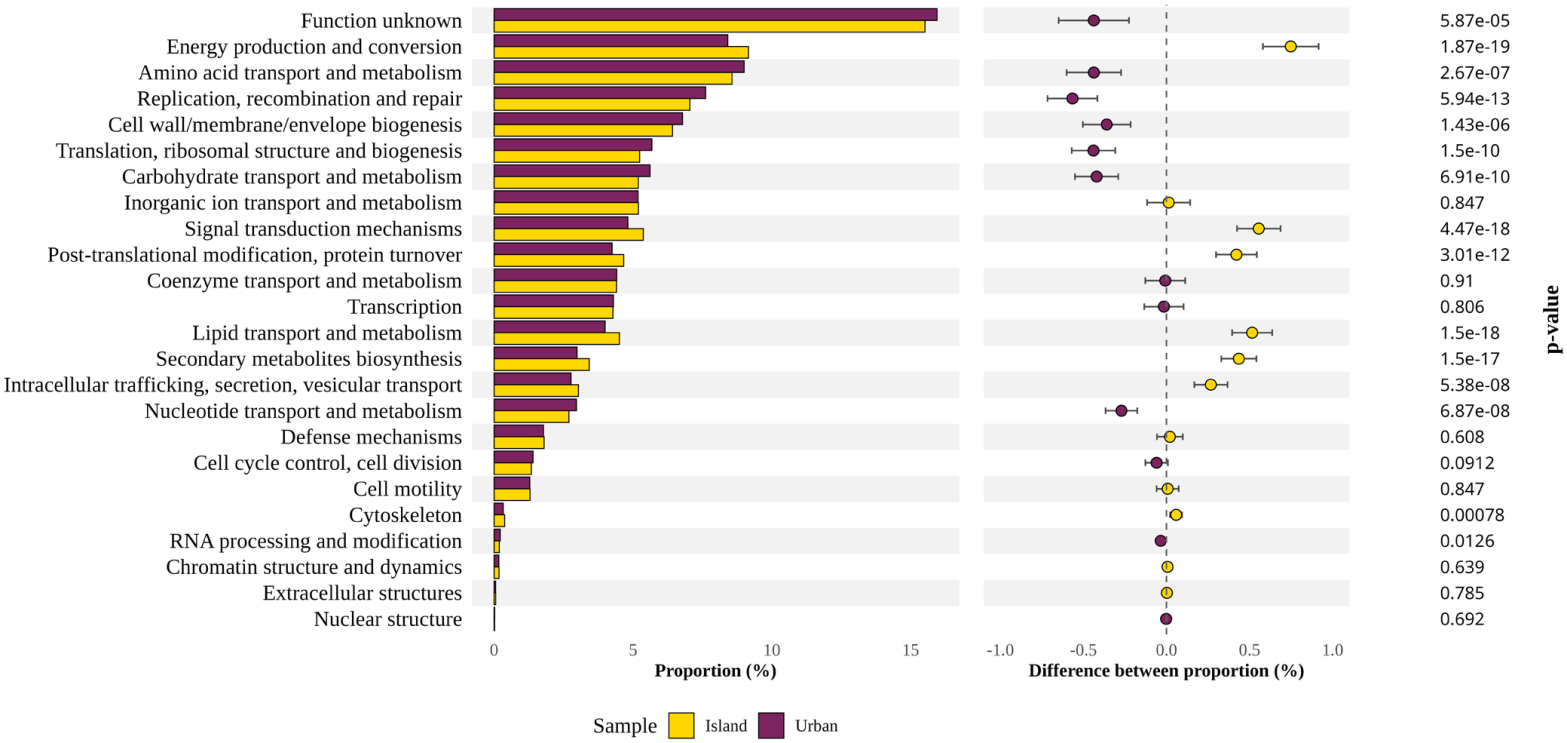
Functional profiling of microbial communities based on Clusters of Orthologous Genes (COGs). The bar chart represents the relative proportion (%) of metabolic pathways in island and urban microbiomes, alongside the difference between proportions. Statistical significance for each functional category is displayed on the right as precise probability (*p*) values. Sample size: *N* = 10 (5 urban, 5 island).

Functional profiling of the microbial communities was conducted using Clusters of Orthologous Genes (COGs) to evaluate the relative abundance of metabolic pathways (Figure 8). The analysis revealed highly significant differences in the functional potential between the two environments. The island beaches exhibited a significantly higher relative proportion of genes associated with ‘Energy production and conversion’ (*p* = 1.87e‐19), ‘Signal transduction mechanisms’ (*p* = 4.47e‐18), ‘Lipid transport and metabolism’ (*p* = 1.5e‐18), ‘Secondary metabolites biosynthesis’ (*p* = 1.5e‐17) and ‘Post‐translational modification, protein turnover’ (*p* = 3.01e‐12).

In contrast, the urban beach communities were significantly enriched in categories such as ‘Amino acid transport and metabolism’ (*p* = 2.67e‐07), ‘Replication, recombination and repair’ (*p* = 5.94e‐13), ‘Cell wall/membrane/envelope biogenesis’ (*p* = 1.43e‐06), ‘Translation, ribosomal structure and biogenesis’ (*p* = 1.5e‐10), ‘Carbohydrate transport and metabolism’ (*p* = 6.91e‐10) and ‘Nucleotide transport and metabolism’ (*p* = 6.87e‐08). Additionally, several fundamental functional categories, including ‘Inorganic ion transport and metabolism’ (*p* = 0.847) and ‘Transcription’ (*p* = 0.806), displayed no statistically significant differences in their proportions between the two environments.

### 3.6 Genomic Potential for Antibiotic Resistance Genes (ARGs) and Xenobiotics Degradation

**FIGURE 9:**
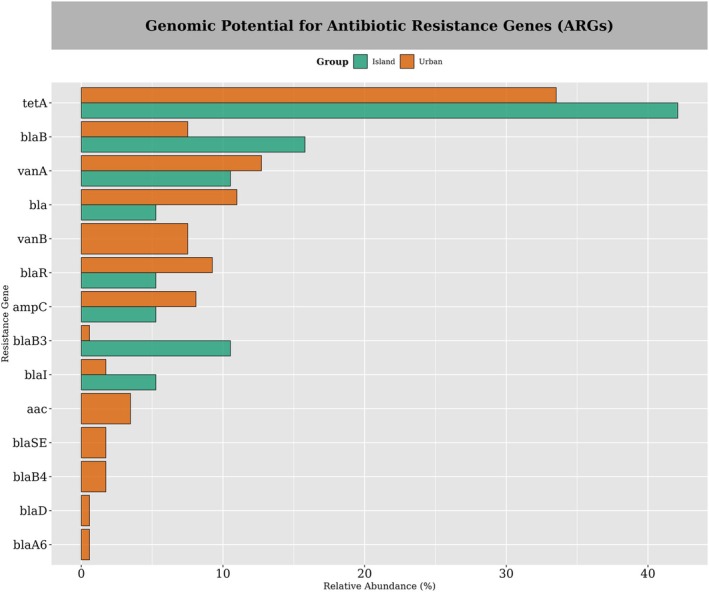
Genomic potential for antibiotic resistance genes (ARGs) across island and urban beaches. Data are presented as the relative abundance (%) of specific resistance genes within the metagenomic profiles. Sample size: *N* = 10 (5 urban, 5 island).

The genomic potential for antibiotic resistance was assessed by analysing the relative abundance of ARGs (Figure 9). The profiles distinctively varied between the two environments. Island beaches were characterised by a highly dominant relative abundance of the *tetA* gene, alongside *blaB* and *blaB3*. In contrast, urban beaches exhibited a broader diversity of prominent resistance genes, displaying higher relative abundances for *vanA*, *bla*, *vanB*, *blaR* and *ampC* compared to the island samples.

**FIGURE 10:**
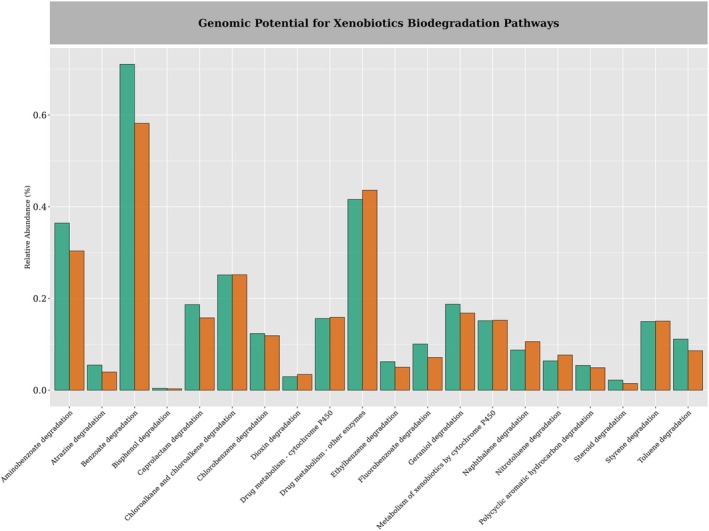
Genomic potential for xenobiotics biodegradation pathways in island and urban microbiomes. Data are presented as the relative abundance (%) of metabolic pathways involved in the degradation of complex compounds. Sample size: *N* = 10 (5 urban, 5 island).

Furthermore, an assessment of xenobiotics biodegradation pathways (Figure 10) indicated that both environments possess the genomic potential for degrading various complex compounds. The relative abundance of these pathways showed variations, with benzoate degradation being the most prominent pathway observed across both groups, followed by aminobenzoate degradation and the metabolism of xenobiotics by cytochrome P450.

## 4 Discussion

### 4.1 Physical Chemical Parameters

The analysis of physicochemical parameters revealed marked differences between the samples from the urban and insular areas, with electrical conductivity standing out in particular. Average values reached 416.2 μS/cm in the urban area and 1516.4 μS/cm in the insular area. This substantial difference may be associated with a higher concentration of dissolved ions, notably sodium (Na^+^) and chloride (Cl^−^), whose levels were also higher in the insular samples (Na^+^ = 371.9 mg/L; Cl^−^ = 458.0 mg/L) compared to the urban samples (Na^+^ = 201.0 mg/L; Cl^−^ = 237.3 mg/L). The higher salinity observed in the insular region (0.74 psu) reinforces this association with the increased presence of dissolved ions, directly influencing the water's conductivity.

Overall, the physicochemical profile of the sampled waters suggests good environmental quality, with pH, dissolved oxygen, turbidity and total dissolved solids falling within ranges consistent with balanced aquatic environments and in compliance with national sanitary standards. However, it is important to emphasise that the exclusive assessment of these parameters is not sufficient to determine water potability. Complementary microbiological and specific chemical contaminant analyses are essential, as recommended by guidelines for water quality monitoring.

### 4.2 Microbial Community Structure

As observed in the alpha diversity measures (Figure 4), the presence of some outlier samples suggests variability in microbial community structure within each group, potentially related to specific environmental factors affecting each sample. The high alpha diversity observed in urban samples may reflect the influence of multiple contamination sources and greater environmental heterogeneity. Comparing the samples collected from different locations (Island vs. Urban), the alpha diversity metrics evaluated distinct aspects of richness and evenness in microbial communities, providing a comprehensive view of the diversity in the studied beaches. These findings indicate that the urban environment harbours a more diverse and balanced microbial community compared to the island environment, likely due to increased environmental complexity and multiple sources of microbial influx.

The dispersion of samples in the RDA ordination plot (Figure 6) suggests that different environmental factors may modulate microbial diversity in each setting (Jiang et al. 2021). According to the ordination, variables such as dissolved oxygen and temperature showed strong correlations with urban samples, while pH, salinity and turbidity emerged as key factors influencing island samples. This reinforces the idea that the physicochemical characteristics of water play a fundamental role in structuring microbial communities (Garg and Ateeq 2025). These findings are visually supported by the distribution of samples and environmental vectors in the RDA ordination plot.

The PCoA analysis based on Bray–Curtis distance (Figure 5) confirmed these findings, showing that some sampling points share similarity patterns but cluster into distinct groups due to specific factors, such as differences in contamination sources and water chemical composition. These differences may be associated with specific environmental factors, such as water quality, the presence of pollutants, variations in the hydrological regime and anthropogenic influence. Thus, beta diversity reinforces the existence of ecological niches with distinct microbial communities (Plantinga and Wu 2021; Simon et al. 2024; Xie et al. 2024). The clear separation between the microbiomes suggests that the two environments host distinct microbial communities shaped by different ecological pressures. However, while these ecological patterns are strongly supported by our data, they should be interpreted with caution, given the relatively small sample size (*n* = 10) of the current study, which restricts statistical power. Future studies with expanded spatial and temporal sampling would be valuable to further validate these anthropogenically driven shifts.

As shown in the taxonomic profiles (Figures 2 and 3), Pseudomonadota was the most abundant phylum, frequently associated with aquatic environments characterised by nutrient variability and exposure to contamination sources (Liang et al. 2023). The presence of Bacteroidota was also notable, suggesting a key role in the breakdown of complex polymers and organic residues present in the water column and sediments (Manal Chrairi et al. 2024). Bacillota were more frequently found in samples from sites with higher organic matter deposition, potentially indicating eutrophic conditions. The presence of these taxa supports the hypothesis that certain sampling sites are influenced by anthropogenic nutrient sources, such as domestic wastewater discharges (Filippidou et al. 2016; Sanz‐Montero et al. 2019; Liang et al. 2020; Ge et al. 2024; Mo et al. 2024). In stark contrast, the island environment was massively dominated by Cyanobacteriota, particularly the genus *Prochlorococcus*, which are typical primary producers in oligotrophic, unpolluted marine ecosystems. This distinct difference further underscores the shift from a natural phototroph‐driven community to a heterotroph‐dominated community driven by urban nutrient inputs.

### 4.3 Differential Abundance and Ecological Indicators

To identify the specific biomarkers driving the differences between the two ecosystems, we focused our analysis at the genus level, avoiding redundant comparisons across higher taxonomic ranks. As demonstrated by the differential abundance analysis (Figure 7), urban beaches exhibited a significant enrichment of specific genera, such as *Comamonas*, *Paenibacillus* and *Clostridium*. This suggests that urban beaches are more strongly associated with contamination sources (Ryan et al. 2022) and increased organic matter availability, promoting the proliferation of taxa adapted to eutrophic conditions. For instance, the dominance of groups like *Comamonas* in urban samples may reflect higher pollution levels (Antonelli et al. 2024), as members of these taxa are known for their ability to degrade complex organic compounds. Furthermore, bacteria indicative of environmental contamination were identified, including *Clostridium*, which is known for its pathogenic potential and association with human and animal faecal sources (Braz et al. 2020). The detection of these taxa underscores the importance of microbiological monitoring to evaluate the safety of beaches for human recreation. Additionally, the presence of versatile taxa contributing to elemental cycling indicates that essential ecological functions are still maintained despite anthropogenic impacts (Dang et al. 2008).

In contrast, the dominant taxa in insular beaches are characteristic of less disturbed marine ecosystems. The island environment favoured the enrichment of distinct genera such as *Burkholderia* and *Bacillus* (Figure 7), while being massively dominated by photosynthetic microorganisms like *Prochlorococcus* (Figure 3). This suggests that these insular areas provide more favourable conditions for primary production and remain less impacted by human activities (Tavares et al. 2020).

Overall, these analyses suggest that the microbial composition of the beaches of Belém is complex, reflecting an interplay between natural ecological processes and human‐induced disturbances (Albuquerque et al. 2018). The variation in the relative proportions of specific taxonomic groups highlights the influence of factors such as proximity to urban areas and sources of organic contamination. These taxonomic distribution patterns between urban and insular beaches reinforce the idea that environmental factors, including pollution and nutrient availability, play a fundamental role in shaping microbial community composition (Lin et al. 2021; Zhang et al. 2024).

### 4.4 Functional Potential and Metabolic Adaptations

Beyond taxonomic composition, shotgun metagenomics allowed us to assess the genomic potential of these microbial communities, revealing distinct metabolic strategies between the two environments. As evidenced by the functional profiling of COGs (Figure 8), the island microbiome exhibited a significantly higher relative proportion of genes associated with energy production and conversion. This aligns with the massive dominance of photoautotrophic genera like *Prochlorococcus*, suggesting an ecosystem emphasising metabolic stability, primary production and natural nutrient cycling processes (Sharpe et al. 2023).

In contrast, the urban beach communities were significantly enriched in functional categories related to cell wall biogenesis, amino acid transport and replication processes (Figure 8). This functional shift reflects a microbiome adapted to eutrophic conditions, where high nutrient availability from anthropogenic inputs, such as sewage and organic runoff, drives heterotrophic activity and rapid cell turnover. Furthermore, urban samples exhibited a broader diversity of prominent antibiotic resistance genes (ARGs), notably *vanA*, *blaR* and *ampC* (Figure 9). The accumulation of these resistance determinants indicates that the urban microbiome is heavily influenced by the horizontal gene transfer of resistance mechanisms, a common adaptive response in environments subjected to pollutant runoff and industrial waste (Santos et al. 2021; Singh et al. 2022).

Despite these anthropogenic impacts, the genomic potential for xenobiotics biodegradation pathways was identified in both environments (Figure 10), with benzoate degradation being highly prominent. The robust presence of these degradation pathways, particularly in urban areas enriched with metabolically versatile taxa, highlights the critical role of these microbial communities as biodegraders. These communities act as environmental sentinels, capable of processing complex organic pollutants and mitigating the impact of anthropogenic contaminants in coastal ecosystems (Zhao et al. 2022; Miglani et al. 2022).

Supporting these functional observations, specific taxonomic indicators reflect these distinct ecological strategies. For instance, the genus Candidatus *Pelagibacter* (SAR11 clade) was prominently found in the island samples (Figure 3). As an extremely abundant and streamlined marine bacterium, *Pelagibacter* thrives in oligotrophic waters, where it plays a key role in the carbon and sulphur cycles. Its presence in insular environments reaffirms the low‐nutrient, stable conditions present in these settings, contrasting sharply with the eutrophic conditions of urban beaches (Carini et al. 2012).

Conversely, a notable taxon strongly enriched in urban samples was the genus *Paenibacillus* (Figure 7). Members of this group are known for their spore‐forming ability (Galperin 2016), degradation of recalcitrant organic substrates and antimicrobial production. Their significant enrichment suggests resilience to harsh environmental fluctuations and possibly a link to terrestrial contamination (Eltokhy et al. 2021). These organisms are frequently isolated from environments impacted by organic waste or sewage, supporting the hypothesis of higher anthropogenic impact in urban beach areas. Additionally, some *Paenibacillus* species possess nitrate reduction and denitrification capabilities, further indicating their involvement in nitrogen transformation processes (Behrendt et al. 2010).

Ultimately, these patterns reflect two distinct microbial ecological strategies shaping the coastal environments: (1) Island: specialisation, phototrophy (e.g., *Prochlorococcus*), metabolic stability and natural nutrient cycling. (2) Urban: generalism, xenobiotic metabolism, resistance traits and plasticity.

The confirmed presence of reliable microbial indicators—such as *Comamonas*, *Clostridium* and *Paenibacillus*—in urban beaches validates the exposure to anthropogenic inputs and potentially pathogenic sources. This reinforces the critical importance of continuous microbiological water quality monitoring in public coastal areas to safeguard environmental health.

## 5 Conclusion

This study revealed distinct taxonomic and functional differences between microbial communities from urban and insular beaches, driven by varying degrees of anthropogenic impact. Taxonomically, while both environments were dominated by Pseudomonadota and Bacteroidota, urban beaches exhibited a significant enrichment of genera associated with eutrophic and contaminated habitats, such as *Comamonas*, *Paenibacillus* and *Clostridium*. In contrast, insular beaches were massively dominated by *Prochlorococcus*, a taxon typical of oligotrophic and stable marine ecosystems. Functionally, shotgun metagenomics revealed that urban microbiomes display an adaptive profile, with strong enrichment in pathways related to xenobiotic degradation, antibiotic resistance (e.g., *vanA*, *ampC*, *blaR*) and rapid cellular turnover, reflecting the influence of organic pollution and nutrient loads. Conversely, insular microbiomes exhibited metabolic specialisation, prioritising pathways focused on energy production, signal transduction and secondary metabolite biosynthesis. These findings underscore the importance of preserving insular environments as stable ecological baselines and demonstrate how urban coastal environments serve as hotspots for the selection of metabolically versatile and resistant microorganisms.

## Author Contributions

**Bruno Santana Carneiro:** data curation, formal analysis, visualization, investigation. **Artur Luiz da Costa da Silva:** supervision, project administration, resources, funding acquisition, writing – review and editing. **Adan Rodrigues de Oliveira:** formal analysis, visualization. **Adonney Allan de Oliveira Veras:** data curation, formal analysis, visualization. **Diego Assis das Graças:** conceptualization, data curation, formal analysis, writing – original draft, writing – review and editing, project administration, supervision, validation, funding acquisition, resources, methodology, investigation, visualization, software. **Rafael Azevedo Baraúna:** writing – review and editing, conceptualization, visualization. **Joelma Barbosa de Carvalho:** formal analysis, visualization. **Angelo Felipe Barbosa de Oliveira:** conceptualization, data curation, formal analysis, visualization, writing – original draft, writing – review and editing, supervision, investigation, methodology, software, validation, project administration.

## Funding

This work was supported by Conselho Nacional de Desenvolvimento Científico e Tecnológico (445350/2024‐5).

## Ethics Statement

The authors have nothing to report.

## Conflicts of Interest

The authors declare no conflicts of interest.

## Data Availability

All gene sequences have been deposited at SRA under the BioProject accession number PRJNA1255235. The custom R scripts and the analytical pipeline used to generate the results and figures in this study are available at https://github.com/oliv‐angelus/metagenomic‐R‐pipeline.git.
